# Are extinction opinions extinct?

**DOI:** 10.7717/peerj.3663

**Published:** 2017-08-11

**Authors:** Tamsin E. Lee, Clive Bowman, David L. Roberts

**Affiliations:** 1 Mathematical Institute, University of Oxford, Oxford, UK; 2 Durrell Institute of Conservation and Ecology, School of Anthropology and Conservation, University of Kent at Canterbury, UK

**Keywords:** Extinction model, Data quality, Possibly extinct, IUCN Red List, Sighting uncertainty, Panthera leo, Tachybaptus rufolavatus, Pterodroma caribbaea, Aplonis pelzelni

## Abstract

Extinction models vary in the information they require, the simplest considering the rate of certain sightings only. More complicated methods include uncertain sightings and allow for variation in the reliability of uncertain sightings. Generally extinction models require expert opinion, either as a prior belief that a species is extinct, or to establish the quality of a sighting record, or both. Is this subjectivity necessary? We present two models to explore whether the individual quality of sightings, judged by experts, is strongly informative of the probability of extinction: the ‘quality breakpoint method’ and the ‘quality as variance method’. For the first method we use the Barbary lion as an exemplar. For the second method we use the Barbary lion, Alaotra grebe, Jamaican petrel and Pohnpei starling as exemplars. The ‘quality breakpoint method’ uses certain and uncertain sighting records, and the quality of uncertain records, to establish whether a change point in the rate of sightings can be established using a simultaneous Bayesian optimisation with a non-informative prior. For the Barbary lion, there is a change in subjective quality of sightings around 1930. Unexpectedly sighting quality increases after this date. This suggests that including quality scores from experts can lead to irregular effects and may not offer reliable results. As an alternative, we use quality as a measure of variance around the sightings, not a change in quality. This leads to predictions with larger standard deviations, however the results remain consistent across any prior belief of extinction. Nonetheless, replacing actual quality scores with random quality scores showed little difference, inferring that the quality scores from experts are superfluous. Therefore, we deem the expensive process of obtaining pooled expert estimates as unnecessary, and even when used we recommend that sighting data should have minimal input from experts in terms of assessing the sighting quality at a fine scale. Rather, sightings should be classed as certain or uncertain, using a framework that is as independent of human bias as possible.

## Introduction

The quality of sighting records of rare species, and particularly those that are approaching extinction, vary considerably. This can lead to confusion, particularly when identifying whether a species is extinct, or identifying when a species went extinct (Roberts et al., 2010). An extinction date for a given species is usually inferred from the rate of sightings by assuming that the rate changes after the point of extinction. Recent models (Solow et al., 2012; Thompson et al., 2013, Lee, 2014; Lee et al. 2014 and Jarić & Roberts, 2014) incorporate uncertain sightings as well, thus recorded sightings might occur after extinction. A review of using sightings records to infer extinction is provided by Boakes, Rout & Collen (2015).

Generally sightings are either grouped as certain or uncertain records by researchers, e.g the ivory-billed woodpecker (Solow et al., 2012). The subjective quality rather than the certainty of a record has been less investigated. To incorporate the varying quality of sighting records, Thompson et al. (2013) and Lee (2014) present a method which allows several different classes of uncertain records, where the classification is determined by subjective quality. Their method is optimal if at least one sighting from each coarsely defined group occurs before the last certain sighting. Any approach requires expert information about the quality of records. Suppose we have high resolution information for the quality of sighting records, that is, we have pooled expert opinions on the quality of each individual sighting record. Does expert information actually improve our inference on extinction estimates? We use the Barbary Lion as an initial test-bed. Collecting pooled expert opinions on individual sighting records is a time-expensive exercise, thus only the Barbary lion sightings currently have this high level of detail. Indeed the primary motivation for this paper is to ascertain whether this cost is necessary.

Lee et al. (2015) provided distributions for 32 alleged sightings of the Barbary lion (*Panthera l. leo*) which occurred between 1895 and 1956 in Algeria and Morocco. In this paper we use the individual quality score provided by Lee et al. (2015). We also examine the importance of the expert’s prior of the lion being extinct on the results.

The work of Lee et al. (2015) provides several distributions for each lion sighting. One method considers the expert estimates for three different questions relating to the distinguishability of the species, observer competence and verifiability, and pools across experts and questions linearly, while another pools them logarithmically. The distributions from pooling across experts and questions provide a quality distribution for each sighting, which we use in this paper. For clarity, we present results from using the linear pooling distributions only, since, as can be seen from Lee et al. (2015), the distributions are similar, and thus our conclusions will be similar.

We begin by examining these distributions (‘Examining sighting quality’), where it is already implied that individual distributions for the quality of each sighting may lead to counter-intuitive results, and thus expert opinion on an individual sightings should be ignored. However, before confirming this contentious statement, we incorporate these distributions into an existing extinction model to further understand the effect of sighting quality scores on extinction estimates.

For the lion, we consider two methods to include the additional information about the quality of uncertain sightings. Both these methods are extensions of the Bayesian model of Lee et al. (2014), which assumes a constant population prior to extinction. The first method looks for a breakpoint in the sighting quality, where one would assume that the average sighting quality before extinction is higher than the average sighting quality after extinction (when all sightings should be false). The assumption is that this breakpoint broadly coincides with the change in sighting rate. Quality should inform when all sightings must be false, and vice versa. We refer to this method as the ‘quality breakpoint method’. Alternatively, we use the sighting quality as a measure of uncertainty around a sighting record. We refer to this method as the ‘quality as variance method’. To further explore the effect of quality under this method, we also assign random quality measures, such that if results from simulated random quality measures are similar to the results from actual quality measures, then expert quality measures are superfluous.

To further demonstrate our methods on additional data sets we also consider three birds, the Alaotra grebe, Jamaican petrel and Pohnpei starling. Since there are not quality distributions for the individual sightings, for these three birds we use the uniformly distributed sighting qualities provided by Birdlife International (Lee et al., 2014). There are fewer uncertain sightings with the bird species, disqualifying them as a critical tests of the change point method (see Table 1). For the three bird species, only the ‘quality as variance method’ is applied. As with the lion, the model is also run with random quality measures to determine the importance of quality estimates.

**Table 1:** Sighting data for three bird species. A quality score of 1 indicates a certain sighting.

Alaotra grebe		Jamaican petrel		Pohnpei starling	
Year	Quality	Year	Quality	Year	Quality
1929	1	1789	0.4–0.8	1930	1
1947	0.4–0.8	1829	1	1995	1
1960	1	1847	0.4–0.8	2008	0.4–0.8
1963	1	1866	1		
1969	1	1879	1		
1970	0.1–0.4	1891	0.8–0.9		
1971	0.1–0.4				
1972	0.6–0.8				
1982	0.6–0.8				
1985	0.6–0.8				
1986	0.1–0.4				
1988	0.1–0.4				

As a small addition, we consider the finding of Lee et al. (2014) that the conclusions may depend upon the prior. If one assumes that the prior of extinction is provided by an expert, then perhaps this influence is welcomed. However in our method, for all four species, we use an non-informative prior (Congdon, 2001) effectively integrating over all possible expert’s views. When inferring extinction for a given species it is recommended to always run a model with an uninformative prior. If an expert prior is provided, an additional model with an uninformed prior allows one to observe the effect of the expert’s opinion.

The framework is presented in ‘Model framework’. Within this section we examine the sighting quality, and identify a change point in sighting quality for the Barbary Lion. In ‘The Choice of Prior’ we examine the choice of the expert’s prior, and discuss the influence it has on the outcome, and hence present an alternative, non-informative, prior. In ‘Results’ we discuss our findings: sighting data that consists of certain and uncertain only is the most reliable. Quality is not strongly informative of extinction.

Before discussing the models, it is illuminating to examine the information in sighting quality **q** itself first. Do any changes make rational sense? We do this with the lion sighting data, looking at the general form of the continuous density assumed for **q** ∈ [0, 1] (where 1 is certain), and whether **q** exhibits a change point over time.

## Examining Sighting Quality

The elicitation in Lee et al. (2015) was not carried out explicitly under a belief of extinction or non-extinction. Five experts offered a best estimate and lower/upper bounds for three different aspects of sighting quality (in an un-blinded manner) for each sighting at time *t*. Lee et al. (2015) use the most straightforward way to represent these three points as a probability density, that is as a triangle density. For simplicity we treat experts as exchangeable, ignore any correlation between the best and lower/upper estimates, and also ignore any correlation between the *j* questions (the differential weights of expert competency does something to adjust for inter-expert correlation as does the exhaustive group elicitation process). The quality density for a given sighting *p*(*Q* = *q*_*t*_) is the result from linear pooling across questions and experts. Note that the distribution resulting from pooling across 15 triangle densities, is not a triangle density. Under the Central Limit Theorem of the sum of identical, independently distributions, one could work in accumulated (normalised) quality measures and thus detect a level change rather than a breakpoint over time as we do herein.

**Figure 1:**
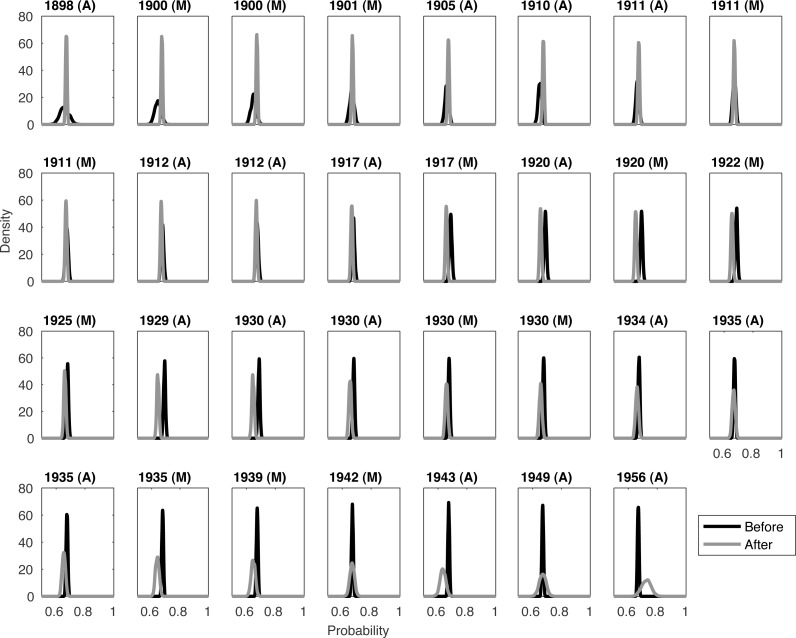
Comparing before and after distributions for the pooled quality densities of each Barbary lion sighting. The year and location (Morocco/Algeria) of the current sighting are listed along the top. The current sighting is grouped with the ‘after’ distribution. The first sighting is 1895 (M). A one-sided *t*-test states that the before and after distributions first become significantly different from each other in 1929.

Examination of the raw *q*_*t*_ values is very noisy. A degree of smoothing is needed to see the choice of right density, any pattern (suggesting *p*(**q**|*E*), where *E* denotes extinction) is or is not equivalent to *p*(**q**|*notE*) and any breakpoint that is informative of extinction. We assume that the first sighting in 1895 (in Morocco) is certain. (Note that no sightings receive a quality score of one, implying no sightings are defined as ‘certain’.) Sweeping across the sightings, sightings are classed as either ‘before’ or ‘after’ the sighting in question, where ‘after’ includes the current sighting. The ‘before’ sightings are combined, as are the ‘after’ distributions, see Fig. 1. Inspection suggests a unimodal distribution like a beta distribution is a sensible choice for the density. A one-sided *t*-test of the quality data in this way indicates that the before and after distributions first become significantly different to each other in 1929.

Notice that the ‘before’ distribution has a large variance when examining the early sightings, and a similar phenomenon for the ‘after’ distributions for the later sightings (Fig. 1). This is because at these extremes, we have less information. For example, when establishing whether extinction occurred between 1895 and 1898 we are comparing the distribution for the single 1895 sighting with the combined distribution from the 31 other sightings.

The issue with this ‘burn-in’ and ‘burn-out’ is evident when examining the peak for the two distributions. Ideally it would be clear to see that the peak (i.e., the mode) of the distribution for before sightings is initially larger, then a switch occurs around 1929. However the lack of data at the time boundaries makes this more challenging to clearly see from the peaks alone. For example, around 1934 the before and after distributions seem very similar. Further smoothing is needed to see any coherent changes.

**Figure 2:**
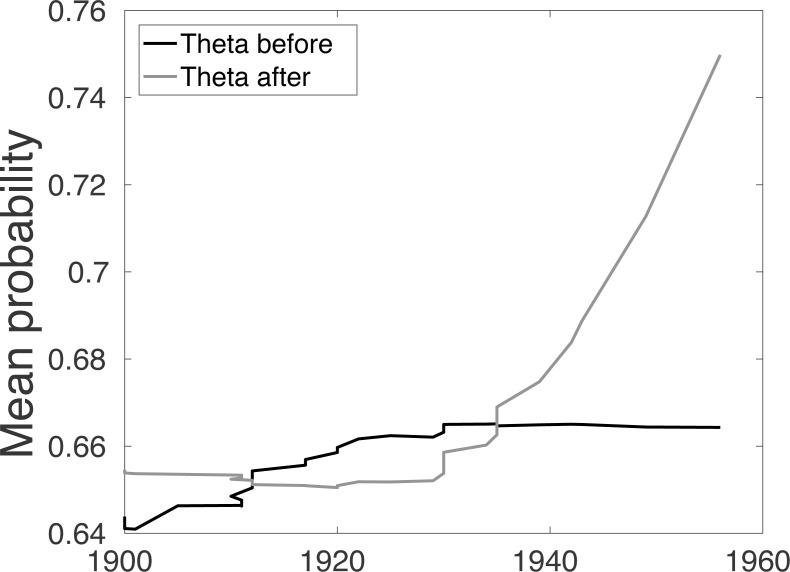
For the Barbary lion data set, the combined average of the sighting record quality before Θ_*b*_(*year*) and after Θ_*a*_(*year*) each sighting.

So instead we consider the combination of the distributions presented in Fig. 1. We denote Θ_*b*_(*year*) as the *peak* of the combined distributions (in Fig. 1) before *year*. And similarly for Θ_*a*_(*year*) from combined distributions (in Fig. 1) after *year*. With this measure it is apparent that a shift in the relationship between these two values occurs around 1930 (Fig. 2), as predicted by the *t*-test.

The Θ_*b*_(*year*) and Θ_*a*_(*year*) allow us to better examine how the quality of sighting changes. The mean of the combined quality of sightings ‘before’ for each sighting year Θ_*b*_(*year*) increases until 1929, and then the quality remains unchanged, Fig. 2. Conversely, the combined ‘after’ distributions, Θ_*a*_(*year*), remains reasonably steady and then increases. This phenomenon is unexpected since one would assume that after extinction, around 1930, the quality of sighting would decrease. However, it is likely that this change is due to human factors such as observers still being alive (first hand account), and the increased use of cameras. This already suggests that the quality breakpoint method may be inconsistent with the sightings process.

Lastly, the empirical Bayes Factor (likelihood ratio) for just the quality data alone (below labelled as *data*_*q*_) is the calculated ratio

(1) \begin{eqnarray*}B{F}_{q}= \frac{\ell ({data}_{q}{|}notExtinct)}{\ell (dat{a}_{q}{|}Extinct)} = \frac{\ell (dat{a}_{q}{|}notE)}{\ell (dat{a}_{q}{|}E)} .\end{eqnarray*}

If the species is extant, the ℓ(*data*_*q*_|*notE*) is small, giving a log Bayes Factor that tends to negative infinity. At each sighting year we calculate the empirical Bayes Factor and find that before 1929 the log Bayes Factor is indeed approximately zero. After 1929 the behaviour of the Bayes Factor changes. We would expect the Bayes Factor to steadily increase after 1929, however, again we observe how human factors have influenced the before and after distributions to create erratic behaviour. Nonetheless, as in the *t*-test there is clearly a shift around 1929 where quality increases. Could this be a technological change?

If the lion data is typical, changes in quality may not indicate the breakpoint for extinction, and thus the sighting quality alone is likely to be unreliable to infer extinction. Let us now consider using it in conjunction with the sighting record. Perhaps, together with analysis on sighting rate, quality scores can provide more information than either of sighting rate or sighting quality alone.

## Model Framework

The objective is to determine the estimated posterior probability distribution of extinction. By Bayes Theorem

(2) \begin{eqnarray*}p(E{|}\mathbf{data})= \frac{l(\mathbf{data}{|}E)\cdot p(E)}{l(\mathbf{data}{|}E)\cdot p(E)+l(\mathbf{data}{|}notE)\cdot p(notE)} \end{eqnarray*}

where *p*(*E*) is the expert’s prior on extinction. Let us retain the general form of the existing problem in Lee et al. (2014). That is, we consider the period of observation (0, *T*) where 0 is the beginning of the sighting record, and *T* is the length of the sighting record. During this observation period, certain and uncertain sightings occur in parallel. The vector **s**_**1**_ represents certain sightings (*s*_1,*t*_) at time *t*, *t* ≤ *T*. Similarly **s**_**2**_ represents uncertain sightings (*s*_2,*t*_) at time *t*, *t* ≤ *T*. Our input **data** comprises of both types of sightings, **s** = **s**_**1**_∪**s**_**2**_. These sightings are used to estimate the posterior probability of extinction and the time at which this extinction occurs. Note that whilst the model does not require an uncertain record **s**_**2**_, a certain record **s**_**1**_ with at least two sightings is required. Certain and uncertain sightings are assumed to follow a stationary Poisson process of regular spacing with constant, unknown rates (*m*_1_ and *m*_2_ respectively). Since we include the possibility of false sightings, sightings may occur after extinction, but at a different constant rate to that rate occurring before extinction. This is an offset denoted *f*_2_ as a background for the whole series (0, *T*). These false sightings by default must only be uncertain sightings. So, given the notation that *l* means likelihood we obtain the four elements of the model:

• *l*(*s*_1_|*notE*) = Bernoulli(1 − *e*^−*m*_1_^)
• *l*(*s*_1_|*E*) = Bernoulli(1 − *e*^−*m*_1_^) if the time is before or equal to the time of extinction, with *l*(*s*_1_|*E*) = 0 if afterwards
• *l*(*s*_2_|*notE*) = Bernoulli(1 − *e*^−*f*_2_−*m*_2_^)
• *l*(*s*_2_|*E*) = Bernoulli(1 − *e*^−*f*_2_−*m*_2_^) if the time is before or equal to the time of extinction, with *l*(*s*_2_|*E*) = Bernoulli(1 − *e*^−*f*_2_^) if afterwards.

The form (1 − *e*^−(⋅)^) is used for efficient parameterisation. We assume no population decline (see Lee et al. (2014) and references therein). This model of Lee et al. (2014) determines a change point in the sighting rate, which provides an estimate for the year of extinction. The input is two sighting records (certain and uncertain) and the output is a probability that the species is extinct at the end of the sighting record, and a corresponding year in which extinction would have occurred. Our method uses: a uniform distribution from the last certain sighting to the end of the sighting record; a non-informative Jeffreys prior (*Beta*(0.5, 0.5)) for non-extinction (Congdon, 2001); together with wide uniform prior distributions (range 0 to 100) for *m*_1_, *m*_2_ and *f*_2_ to ensure that there is no bias.

Now consider that our input **data** comprises of both certain and uncertain sightings, *s* = **s**_**1**_∪**s**_**2**_, and the individual quality of uncertain sightings, **q**. We interpret individual quality scores as a score for the year in which the sighting occurred. However, we require a quality score for every year, even if no sighting occurred. We take a wide interpretation of what quality is. One could infer that a high quality sighting (e.g., a skin sample that can be tested) implies that the sighting is certain (or close to certain), and conversely, a low quality sighting (e.g., a second-hand verbal account) is less certain to be a true sighting. Note that the quality vector **q** initially seems to only have a quality assigned during years of uncertain sightings. Later we will discuss how we assign a sighting quality for all other years. We take quality simply to be a subjective attribute of the sighting—no implicit model of its basis is assumed. A method for eliciting the quality measures is provided in Lee et al. (2015).

Let us partition the **data** into the stochastic sightings, **s**, and the stochastic quality measure for each of the uncertain sightings (**q**), measured and stochastically modelled simultaneously. Note that only **q** ∈ [0, 1) are used as quality measures, which only relate to uncertain sightings. The sighting quality **q** is thus in a sense a nuisance variable which we take as *Beta* distributed, which ensures that it is bounded between [0, 1] as required. We use non-informative *Exponential*(1) and Jeffreys (*Beta*(0.5, 0.5)) priors for its parameters using the Stroud (1994) method in Congdon (2001). Equation (3) in full would rely upon specifying a general stochastic model for the quality measure *p*(**Q** = **q**) under the alternative hypothesis of extinction versus that under non-extinction.

One would expect that after extinction, sighting quality drops, yet ‘Examining Sighting Quality’ proved this may not be the case. So for simplicity (and to avoid specifying even more unknown priors in the computations to integrate over) we assume herein that the probabilistic generating process for quality attribution is unaffected by whether the species is extinct or not, unless the sighting is deemed as ‘certain’. Formally, *l*(**q**|*E*) = *l*(**q**|*notE*) = *l*(**q**). This approximation is reasonable since the alternative requires an estimate for the error process arising from experts assigning quality measures for *l*(**q**|*E*) and *l*(**q**|*notE*), which needs repeated blinded data for a variety of species whose extinction date and status were well known but that investigative adjudicators had little experience about before. A significant practical challenge.

For the quality breakpoint method, we assume that the probabilistic generating process of sightings is unrelated to the generating process of quality attribution, that is, *p*(**data**) = *p*(**s**&**q**) = *p*(**s**)⋅*p*(**q**). The two processes are taken to be independent. So integrating out the uncertainty of the nuisance gives

(3a) \begin{eqnarray*}l(\mathbf{data}{|}E)\rightarrow \int \nolimits \nolimits _{0}^{1}{l}_{s}(\mathbf{s}{|}E)\cdot {l}_{q}(\mathbf{q}{|}E) \mathrm{d}p(\mathbf{q}),\end{eqnarray*}

(3b) \begin{eqnarray*}l(\mathbf{data}{|}notE)\rightarrow \int \nolimits \nolimits _{0}^{1}{l}_{s}(\mathbf{s}{|}notE)\cdot {l}_{q}(\mathbf{q}{|}notE) \mathrm{d}p(\mathbf{q}),\end{eqnarray*}

which are the weighted sums of likelihoods over the stochasticity of the quality measure at **Q** = **q**, where **Q** is a particular realisation of **q**. Conjugacy makes this integration efficient. The likelihoods Eq. (3) can be fed into Eq. (2) to yield the extinction posterior for sightings *p*(*E*|**s**). The same four-part structure of the approach (with subscript 1 indicating certain sightings and subscript 2 indicating uncertain sightings) would now follow but with *Beta* likelihoods for **q**. However, the quality measures are for uncertain sightings only, thus:

• *l*(*q*_2_|*notE*) = *Beta*(*α* + *α*_2_, *β* + *β*_2_)
• *l*(*q*_2_|*E*) = *Beta*(*α* + *α*_2_, *β* + *β*_2_) if the time is before or equal to the time of extinction, with *l*(*q*_2_|*E*) = *Beta*(*α*, *β*) if afterwards.

The overall form for estimation over *s*&*q* is then comparable to that of Lee et al. (2014) and can be fitted using the same OpenBugs (2012) approach via Markov Chain Monte Carlo (MCMC) integration. Any change point in **q** will reinforce or conflict with any change point in **s** in the overall optimistaion for *p*(*E*|**data**). Later work may investigate our assumption that sighting rate and quality scores are independent. For example, perhaps an event occurs (change in IUCN classification or a reward offered) that causes an influx of low quality sightings.

For the quality as variance method we assume that *p*(**data**) = *p*(**s**|**q**)⋅*p*(**q**) and solve accordingly. In this case *p*(**q**) is taken to be a *Gamma* distributed expansion/shrinkage factor to the variance of the rate of the uncertain (only) Poisson distributed sightings **s**_**2**_ above. Full calculation detail is given in a later section. Again, later work may test whether it is appropriate to view (**s**&**q**) as independent (conditional on extinction) and this Bayesian model extended.

One thus chooses between: the changes in sighting quality inform the inference of whether extinction has occurred by directly affecting the likelihood of the sightings (the quality breakpoint method). Or, that quality is a proxy for the variance around the degree of certainty of the uncertain sightings and so affects the likelihood of the sightings indirectly (the quality as variance method). In either case, integrating over the nuisance modifies the relative sightings likelihoods which arise from the simple approach of assuming all sightings are of similar quality used in Lee et al. (2014).

### Bayesian modelling: quality data

Certain and uncertain sightings are interlaced over time, yet for their joint Bayesian modelling a quality measure is needed at each time point for either type of sighting. For implementation on the lion data we assume the first sighting and the most certain sighting (in 1925) are both certain, that is they have a quality score of one and the lion is assumed extant in 1925. The remaining sightings are left as uncertain.

By the nature of the logic of the observing process, there are some *per force* missing quality values. Accordingly a form of Last Observation Carried Forward (LOCF) for uncertain qualities is used to fill in any such missing quality values. However, LOCF is modified such that observations carried forward are randomly drawn from the quality density from the last sighting *p*(*Q* = *q*_*t*_). Furthermore, since the method of Lee et al. (2014) requires a certain sighting at the beginning, and an uncertain sighting may not occur for some years after this initial sighting, there needs to be a quality measure for these unobserved values. As with LOCF, a form of First Observation Carried Backwards (FOCB) is deployed such that the quality for previous years is drawn from the density for the first sighting. Therefore, due to our modified form of FOCB and LOCF, the quality density of the first uncertain observation is used from the first (certain) sighting until the second uncertain observation, where the mean quality is used at the time of the first sighting. The argument is that whilst the mean from the quality density for the first uncertain sighting is the closest to the unknown quality of ‘never seen’ uncertain sightings during this time period, there is still uncertainty around this value, so using information from the distribution as a whole is more appropriate. Using quality information from the whole time period (not just the quality from the first sighting quality) would be using information from a significantly different time period and induce bias. Of course a more sophisticated stochastic model for missing quality data could be posed.

### Bayesian modelling: quality as a breakpoint method

Exactly the same approach as in Lee et al. (2014) is used in which a change in sighting rate is sought, which infers an extinction time. The model provides a probability and variance around this estimate, that is, the probability that the species is extinct, and the variance around this probability.

Now due to the independence of **s** and **q**, simply a second simultaneous Bayesian optimisation of a beta distributed quality variable over time is made around a common extinction point with the sightings. A non-informative hyper-prior is used. For demonstration purposes, here the model is run on the data as it stands every year after the last certain sighting, up to 2016. This allows us to examine the effect of additional uncertain sightings.

For each run of the model, the probability that the lion is extinct (the posterior) and the standard deviation around this estimate is noted. When using the data set as it stood in 2016, we also note the corresponding inferred extinction time. We also make a note of the corresponding inferred extinction time when the posterior first overwhelms the experts’ prior belief of extinction.

### Bayesian modelling: quality as variance method

Let us return to the Bayesian model of Lee et al. (2014). The rate of uncertain sightings is assumed to follow a Poisson distribution with rate *m*_2_ + *f*_2_ where *m*_2_ is the rate of true uncertain sightings and *f*_2_ is the rate of false uncertain sightings, such that a change point indicates when *m*_2_ sightings have ceased, that is, extinction has occurred. The rate of certain sightings *m*_1_ is consistent until extinction.

The same model and computational algorithm as in Lee et al. (2014) is used. No attempt is made to model an exponentially declining quality after the extinction time - rather a common offset is used over all cases. Unlike Lee et al. (2014), here we use vague parameter priors throughout. The sensitivity of the initial prior on being extant is explored with the lion species only, since the effect of the prior on the bird species data sets has already been explored by Lee et al. (2014).

Instead of seeking a breakpoint in the quality **q**, we slightly relax the assumption that quality and sightings are independent. Whilst we maintain that the occurrence of sightings, and the quality of sightings are independent, we now incorporate the quality of a sightings as unique variance around each sighting. Quality then behaves like a fractional replication factor. To incorporate quality, each uncertain sighting follows a distribution with expected rate as before but with a variance that increases as the quality of the sighting decreases. So an uncertain observation at time *t* is ‘fuzzed’ by a Gamma distribution, such that the ‘fuzzed’ rate of uncertain sightings is

(4) \begin{eqnarray*}{m}_{2}^{{^{\prime}}}={m}_{2}r,\end{eqnarray*}

where *r* is a random variable. The random variable *r* is drawn from a Gamma distribution of mean 1

(5) \begin{eqnarray*}r\sim \Gamma (1/{H}_{t},1/{H}_{t}),\end{eqnarray*}

where *H*_*t*_ =  − *a*ln(*q*_*t*_), *q*_*t*_ is the corresponding beta distributed (0, 1] quality score for the sighting, and *a* is a penalty factor such that large *a* penalises low quality sightings more. In doing this we are using the log link model (McCullagh & Nelder, 1989) philosophy. In this, *a* = 1, 2, 4, where *a* is a penalty variable which quantifies the relationship between the measured quality and the ‘fuzz’ applied to the model. Large values of *a* model when a small change in quality produces with a large uncertainty i.e., uncertainty is inflated. If sighting quality is not considered important, *a* is small such that at *a* = 0 and it reverts back to the model of Lee et al. (2014).

Equations (4) and (5) ensures: the rate remains positive; the variance around uncertain sightings is −*a*ln(*q*_*t*_); and when sightings are certain (*q*_*i*_ = 1), the rate is not fuzzed, ${m}_{2}^{{^{\prime}}}={m}_{2}$. Note that the variance is not added to *f*_2_, the false sightings offset. Under this adaptation of the model, *f*_2_ can be thought of us a constant characteristic background rate of sightings for the whole data set that remains unchanged throughout the whole period—an attribute which changes solely from species to species.

As with the quality as a breakpoint method, the model is run on the data as it stands every year after the 1925 sighting. For each run of the model, the probability that the lion is extinct (the posterior) and the standard deviation around this estimate is noted. When using the data set as it stands in 2016, we also note the corresponding inferred extinction time, and we make a note of the corresponding inferred extinction time when the posterior first overwhelms the experts’ prior belief of extinction. In addition, we run this model on the three bird species. The same output details are recorded as with the lion species.

## The Choice of Prior

**Figure 3:**
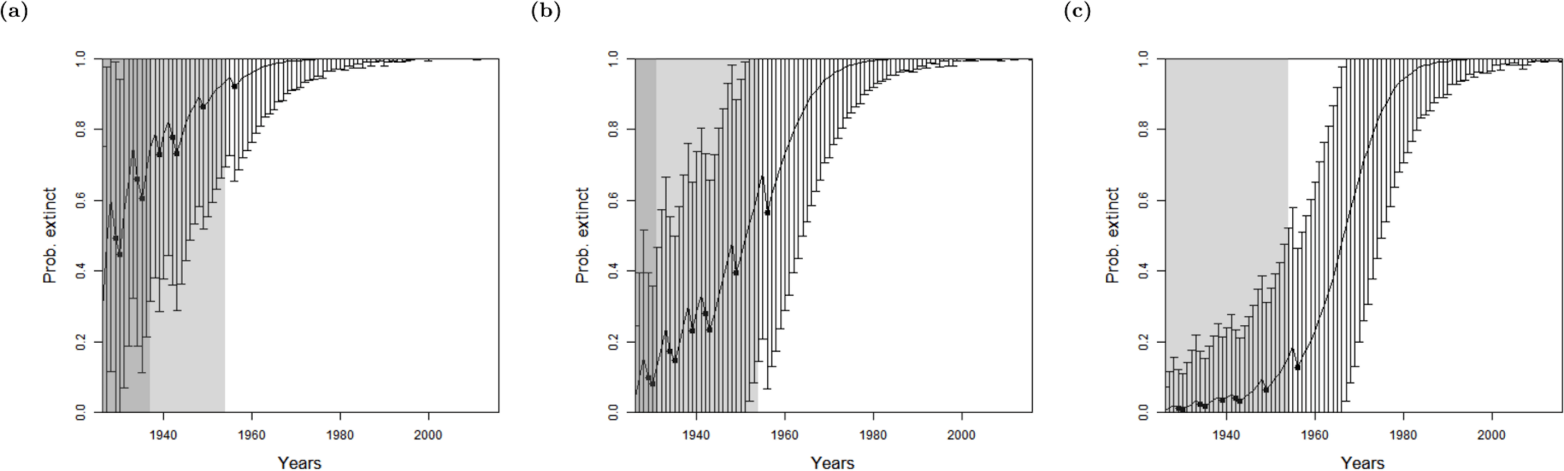
Results for the Barbary lion from the method of Lee et al. (2014), where sightings are divided into certain and uncertain over a varying expert’s value for prior of extinction: (A) 0.9 (B) 0.5 (C) 0.1. The bars represent the standard deviation around the estimate and the black circles indicate when an uncertain sighting occurred. Note changes to the standard deviation of estimates as well as the curve translocation. The light shaded region marks the predicted extinction year identified by the model in 2016 (using the full dataset), which is in 1954 in all cases. The dark shaded region marks the predicted extinction year identified when the likelihood first overwhelms the prior, i.e., the extinction date inferred when there is enough data, which is in 1953 in all cases, giving a prediction extinction year of (A) 1937 (B) 1931 (C) 1924 (before the last certain sighting of 1925 so is clearly incorrect).

As previously shown, the method of Lee et al. (2014) is affected by the experts’ prior. This phenomenon is presented for the bird data sets in Lee et al. (2014), and with the Barbary lion data set in Fig. 3. Whilst the three different choices of prior are all initially overwhelmed by the posterior in 1953 (that is, the extinction probability is larger than the prior), the estimated extinction year that corresponds with the data set as it stood in 1953 is different. When the model is run with the data that exists up to 1953 only, with a prior belief of extinction of 0.9, the model infers extinction occurred in 1936; with a prior of 0.5, the model infers extinction occurred in 1931; and with a prior of 0.1, the model infers extinction occurred in 1924, which is before the ‘certain’ sighting of 1925 occurs, so is clearly incorrect.

Using the full data set as it stands in 2016 only includes one additional sighting after 1953, a sighting in 1956. The estimated extinction year does not vary between the three prior choices: a prior belief of extinction of 0.9, 0.5 and 0.1 all predict extinction in 1954. Therefore the model assumes that the 1956 sighting is false. Additionally, the results are less influenced by the prior when the model is run with more data, as one would expect.

**Figure 4:**
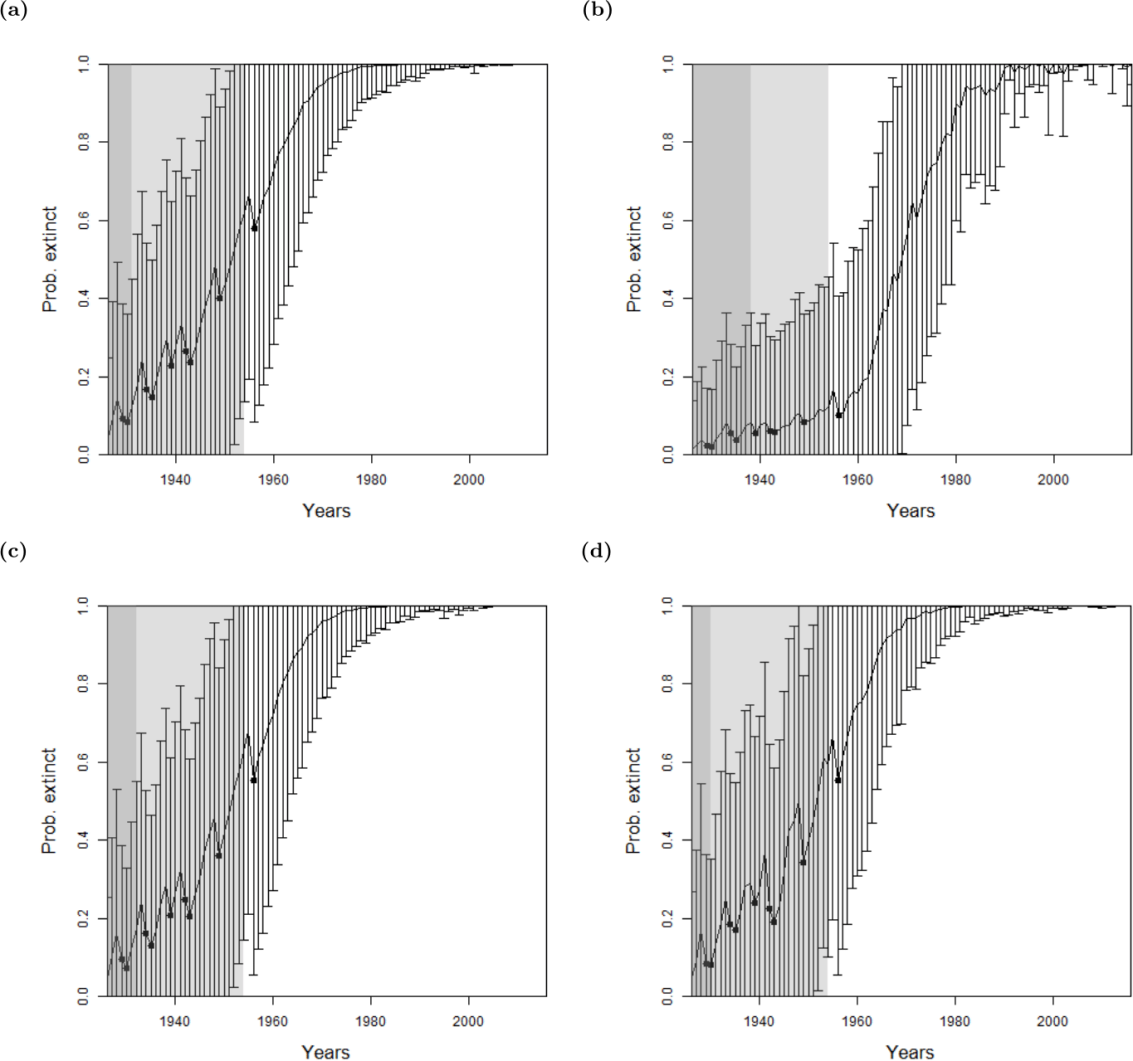
The probability of extinction for every year after the last certain sighting (using data only to that particular year) for the Barbary lion from (A) the method of Lee et al. (2014) where sightings are divided into certain and uncertain now with an non-informative hyper-prior of extinction, (B) the ‘quality break point method’, the ‘quality as a variance method’, where the quality is either (C) provided by experts, or (D) the quality is random. The bars represent the standard deviation around the estimate and the black circles indicate when an uncertain sighting occurred. The light shaded region marks the predicted extinction year identified by the model in 2016 (using the full dataset), which is in 1954 in all cases. The dark shaded region marks the predicted extinction year identified when the likelihood first overwhelms the prior, i.e., the extinction date inferred when there is enough data, which is in (A, C, D) 1953 and (B) 1969, giving a prediction extinction year of (A) 1878 (B) 1938 (C) 1932 (D) 1930.

To avoid the bias towards the expert’s prior choice, for all four species, we use a non-informative hyper-prior—the beta distribution *p*(*E*) ∼ *Beta*(0.5, 0.5) (Congdon, 2001) and optimise accordingly. This distribution actually slightly favours the extremes equally, when the species is definitely extinct *p*(*E*) = 0 or definitely extant *p*(*E*) = 1, and all other possibilities are equally likely to each other. The expected value of this density is 0.5 i.e., a 50:50 evens bet of prior ignorance. The model from Lee et al. (2014) applied to the lion data set, with this improvement, provides similar results to when a prior of 0.5 is used, as one would expect, see Fig. 4A. The first year that the likelihood overwhelms the average of the prior, 0.5, occurs in 1953 again, which gives a corresponding year of extinction of 1931. When the model is run on the sighting record as it stands in 2016, the corresponding extinction year is 1954. Although these results are similar to a prior of 0.5, a non-informative prior is preferred to a point estimate as it integrates over all uncertainties appropriately.

## Results

Lee et al. (2014) considered the rate of certain and uncertain sightings, and sought to find a change point in the sighting rate, thus the model requires as least two certain sightings. The individual quality of each sighting was not considered. As discussed in the previous section, using this approach the probability that the Barbary lion is extinct is not above the average of the experts’ prior until after 1953 (Fig. 4A), which has a corresponding extinction date estimate of 1931, approximately the same time period that there is a change in the quality of the data (Fig. 2).

When seeking a change in the quality (‘quality as a breakpoint method’), there is low overall information, that is, it is only after an absence of sightings for 13 years after the very last sighting when the likelihood first overwhelms the prior (Fig. 4B). This is inconsistent with the sightings information. The model cannot identify a common change zone for sighting rate whilst simultaneously identifying the change in sighting quality.

Continuing with the lion, we used the sighting quality as a variance method with both expert estimates (Fig. 4C) and random estimates (Fig. 4D) for the sighting qualities. Both choices provide results very similar to the model of Lee et al. (2014), which does not include sighting quality (Fig. 4A). The posterior from all three models first overwhelm the average of the prior in 1953, and at this point, estimate extinction to have occurred around 1931. As before, the effect of the quality is apparent when the full data set up to 2016, is used. The additional sighting in 1956 affects the sighting rate, causing the change point in sighting rate to shift to 1954.

**Figure 5:**
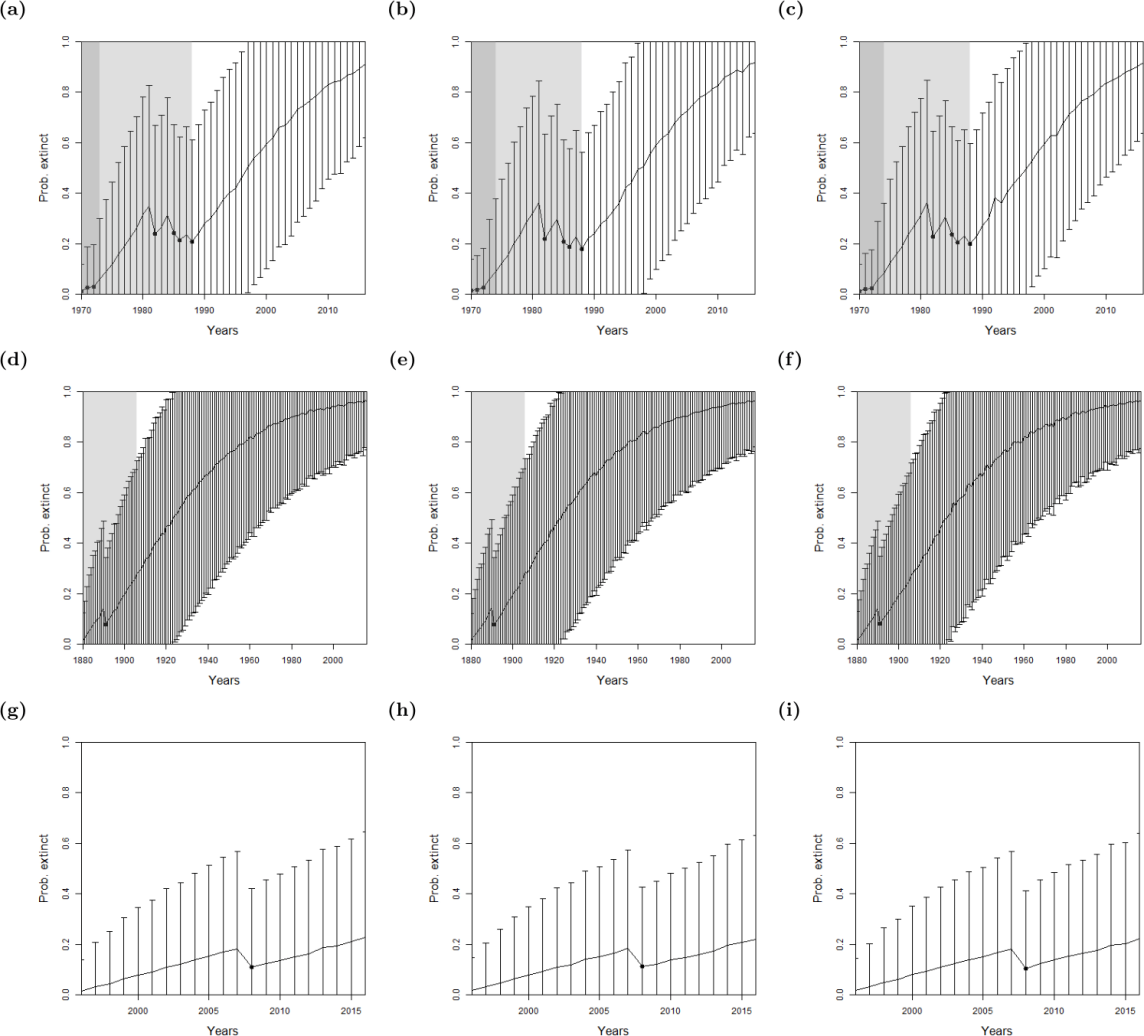
The probability of extinction for every year after the last certain sighting (using data only to that particular year), with the ‘quality as a variance method’, where the quality is either (A, D, G) excluding quality, or using the quality as a variance where the quality is either (B, E, H) provided by experts, or (C, F, I) the quality is random. The bars represent the standard deviation around the estimate and the black circles indicate when an uncertain sighting occurred. All models use a non-informative hyper-prior on extinction. When quality is included, a penalty *a* = 2 is used. The light shaded region marks the predicted extinction year identified by the model in 2016 (using the full dataset). The dark shaded region marks the predicted extinction year identified when the likelihood first overwhelms the prior, i.e., the extinction date inferred when there is enough data. Alaotra grebe (A–C): the likelihood first overwhelms the prior in 1998, with a corresponding extinction year of 1973 (A) or 1974 (B, C). With the full dataset, extinction is estimated to have occurred in 1988 in all three cases. Jamaican petrel (D–F): the likelihood first overwhelms the expected value of the prior in 1925 with a corresponding extinction year of 1878, before the last certain sighting. With the full dataset, extinction is estimated to have occurred in 1906. Pohnpei starling (G–I): the likelihood never overwhelms the prior meaning that there is not enough data.

For the bird species, there were not enough uncertain sightings to seek a change point in sighting data, see Table 1. Therefore, we only use the method where quality is implemented as a variance. As with the lion, we find similar results between omitting sighting quality, using the sighting quality estimates, and using random sighting quality estimates, see Fig. 5. Moreover, the Pohnpei starling demonstrates the challenge of trying to infer an extinction date when there is a paucity of data, since the likelihood never overwhelms the prior. This is a similar problem to the ‘quality breakpoint method’ (Fig. 4B) where a long period of no sightings was required.

Reducing the weighting penalty, *a*, further reduces the effect of the sighting quality, whereas increasing *a* increases the standard deviation around each annual estimate. When *a* is increased to, say, *a* = 10, the change point in the sighting record dissipates into fuzz, and thus cannot be determined. That is, everything becomes uncertain.

For all species, there is a consistent similarity of extinction inferences over all species between using actual quality measures and simulated random quality scores. Since it was shown in ‘Examining Sighting Quality’ that the quality distributions are non-random through time, this points to the lack of sensitivity to expert opinion for the inference of extinction.

## Discussion

We have presented a method that considers the quality of each sighting individually. Previous work has initially used sightings that are classed as either certain or uncertain (Solow et al., 2012; Lee, 2014). Further work then sought to divide uncertain sightings further, into several categories (Lee et al., 2014). However, each additional classification provides less information about the rate of occurrence for this particular class of sighting (law of diminishing returns). To avoid continually dividing uncertain sightings into more specific categories we investigated a method that sought to find the change point in the continuous quality of sightings. This required several experts to rate all sightings of a species. As discussed in previous work (Lee et al., 2015), the method by which the experts are questioned is important to the outcome.

There is a change in sighting quality scores at 1930, where sightings after this date have a perceived higher quality. However the change in sighting quality may be picking up on our preference to believe accounts from living observers more than records left by deceased observers. There is a large literature on unbiased Bayesian elicitation methods to help avoid this (see review article Kuhnert, Martin & Griffiths, 2010). Being blinded to date and the historical age of sightings is important—however, this is difficult. Technological changes over time are apparent even when species observations are not explicitly labelled (an old photo is clearly an old photo). Whilst human nuances affect all sighting records, and thus all extinction models, methods which rely more heavily on expert opinion may be more susceptible to these external factors.

Establishing the balance between an extinction model with assumptions that over simplify, and a model that seeks to incorporate everything, is discussed by Caley & Barry (2014), where the authors develop an extinction model that does not constrict the species population to be constant (as assumed here), nor declining. In line with their findings, our work also suggests that a simple model makes it easier to identify the underlying population processes. If quality is to be used, the quality as variance method is recommended.

We have shown that the rate of sightings is the strongest indicator to infer extinction, and too much information about the quality of the sighting can actually be detrimental. Ideally a sighting record would be a list of certain and uncertain sightings only. Using only these two parallel sighting records, a Bayesian model (Lee et al., 2014 or Thompson et al., 2013), with the non-informative prior presented here, could establish a synchronised change point to infer an extinction date. No further classification, nor prior belief about extinction, is required.

The less propensity for human influence in the sighting data the better. As such, an objective method of assessing whether sightings are certain or uncertain is needed. However, even the rate of sightings is susceptible to human influence, such as periods of high interest due to publicity. Future work could try to quantify the effect of publicity on sighting rate.

##  Supplemental Information

10.7717/peerj.3663/supp-1 Supplemental Information 1 Random values drawn from the distributions for quality of each individual sighting Each column represents a sighting.

10.7717/peerj.3663/supp-2 Supplemental Information 2 R code used to implement the model R code, which uses JAGS, to estimate extinction. Certain and uncertain sightings are used, where quality scores are used as variance around the uncertain sightings.

10.7717/peerj.3663/supp-3 Supplemental Information 3 R code used to implement the model R code, which uses JAGS, to estimate extinction where a unique change point in quality scores, and sighting rates (certain and uncertain) is sought.

## Additional Information and Declarations

### Data Availability

The following information was supplied regarding data availability: The raw data and code have been provided as Supplemental Files.
